# 3D exoscopic *versus* microscopic superficial temporal artery to middle cerebral artery bypass surgery for moyamoya disease – a comparative series

**DOI:** 10.1007/s00701-024-06100-3

**Published:** 2024-06-07

**Authors:** Michael Veldeman, Tobias Rossmann, Ville Nurminen, Justiina Huhtakangas, Roel Hubert Louis Haeren, Ahmad Hafez, Mika Niemela, Martin Lehecka

**Affiliations:** 1 Department of Neurosurgery, University of Helsinki and Helsinki University Hospital, Helsinki, Finland; 2 Department of Neurosurgery, RWTH Aachen University Hospital, Aachen, Germany; 3 Department of Neurosurgery, Neuromed Campus, Kepler University Hospital, Linz, Austria; 4 Maastricht Universitair Medisch Centrum+, Maastricht, Netherlands

**Keywords:** Exoscope, Microscope, Moyamoya angiopathy, Moyamoya disease, Extracranial to intracranial bypass, Superficial temporal artery, Middle cerebral artery, Aesculap Aeos, Zeiss Pentero

## Abstract

**Purpose:**

Superficial temporal artery to middle cerebral artery (STA-MCA) direct bypass surgery is the most common surgical procedure to treat moyamoya disease (MMD). Here, we aim to compare the performance of the 3D exoscope in bypass surgery with the gold standard operative microscope.

**Methods:**

All direct STA-MCA bypass procedures performed at a single university hospital for MMD between 2015 and 2023 were considered for inclusion. Data were retrospectively collected from patient files and surgical video material. From 2020 onwards, bypass procedures were exclusively performed using a digital three-dimensional exoscope as visualization device. Results were compared with a microsurgical bypass control group (2015–2019). The primary endpoint was defined as total duration of surgery, duration of completing the vascular anastomosis (ischemia time), bypass patency, number of stiches to perform the anastomosis, added stiches after leakage testing of the anastomosis and the Glasgow outcome scale (GOS) at last follow-up as secondary outcome parameter.

**Results:**

A total of 16 consecutive moyamoya patients underwent 21 STA-MCA bypass procedures. Thereof, six patients were operated using a microscope and ten patients using an exoscope (ORBEYE® *n* = 1; AEOS® *n* = 9).

Total duration of surgery was comparable between devices (microscope: 313 min. ± 116 vs. exoscope: 279 min. ± 42; *p* = 0.647). Ischemia time also proved similar between groups (microscope: 43 min. ± 19 vs. exoscope: 41 min. ± 7; *p* = 0.701). No differences were noted in bypass patency rates. The number of stiches per anastomosis was similar between visualization devices (microscope: 17 ± 4 vs. exoscope: 17 ± 2; *p* = 0.887). In contrast, more additional stiches were needed in microscopic anastomoses after leakage testing the bypass (*p* = 0.035).

**Conclusion:**

Taking into account the small sample size, end-to-side bypass surgery for moyamoya disease using a foot switch-operated 3D exoscope was not associated with more complications and led to comparable clinical and radiological results as microscopic bypass surgery.

**Supplementary Information:**

The online version contains supplementary material available at 10.1007/s00701-024-06100-3.

## Introduction

The most commonly performed direct revascularization for Moyamoya disease (MMD), is anastomosis of the superficial temporal artery (STA) to distal M3 or M4 branches of the middle cerebral artery (MCA) (STA-MCA bypass) [3, 11, 12]. The aim of direct revascularization is to improve cerebral blood flow (CBF) and prevent either transient ischemic attacks (TIAs) or cerebral infarction [13]. Based on expert consensus, primary treatment of symptomatic MMD via surgical revascularization should be considered [1, 14].

Visualization in cerebrovascular surgery has - since the dawn of microneurosurgery - consisted out of the counterbalanced binocular operating microscope [23]. Three-dimensional (3D) exoscopes offer an alternative for surgical magnification and illumination, with specific advantages related to image quality, ergonomics and training possibilities [4]. First generation exoscopes offer image quality that challenges conventional optics with the additional benefits of higher magnification, live image processing ameliorating tissue differentiation, and a larger depth of field [21].

In an experimental cerebral bypass setting, comparable procedural quality was found for the microscope and the 3D exoscope [9, 10]. Exoscope systems have been assessed in respect to their performance in cerebral revascularization surgery [16, 17], but not in direct comparison with the operating microscope.

The aim of this study was to provide a comparative analysis of the surgical performance of 3D exoscopes in STA-MCA bypass surgery exclusively carried out for MMD *versus* microscope-aided vascular anastomosis. Here, we hypothesized that exoscope use can lead to comparable results regarding overall duration of surgery, duration of end-to-side anastomosis (ischemia time) and bypass patency rates.

## Methods

### Patient population and study design

All patients with MMD in Finland, for which bypass surgery is considered, are assessed at the Helsinki university hospital (HUS). Moyamoya angiopathy and disease are diagnosed based on international criteria and recommendation [1, 8]. The decision to recommend flow-augmentation surgery is made on an individual basis, in interdisciplinary consensus among pediatricians, neurologists and neuroradiologists. Treatment options are considered based on clinical presentation, course of the disease, angiographic severity and evidently, patient age and opinion. If bypass surgery is considered an option, patients undergo six-vessel cerebral angiography to estimate anatomical feasibility (incl. donor size).

Since 2015, all EC-IC procedures in Helsinki, and thereby Finland, were performed by the senior author (ML) with few exceptions i.e. during the Helsinki live courses. Since 2020 all surgeries have been performed with a 3D digital exoscope (ORBEYE^®^*n* = 1; AEOS^®^*n* = 9).

In this study, we aim to compare all STA-MCA bypass procedures for MMD using the surgical microscope (pre-2020) *versus* those procedures using the exoscope (2020–2023). It should be noted that the senior author had extensive prior experience in EC-IC bypass surgery as well as exoscopic microneurosurgery in form of skull base and neurovascular procedures [19, 21].

This article is written in accordance with the Preferred Reporting of Case Series in Surgery (PROCESS) statement for reporting observational studies/case series.

### Surgical treatment

Bypass surgery is subjected to numerous nuances and there exists high variability in surgical technique between surgeons and MMD treating centers. Though the focus of this study does not lie on surgical technicalities, we provide an overview of the STA-MCA procedure as currently practices at HUS.

Whenever surgically feasible, based on donor size and anticipated recipient caliber, a direct in situ bypass is preferred over an indirect bypass (i.e. encoephao-duro-arterio-myo-synangiosis or EDMS). The STA-MCA anastomoses are sometimes combined with EDMS in patients under 25 years of age, but purely indirect bypass procedures are reserved for young children only.

**Fig. 1:**
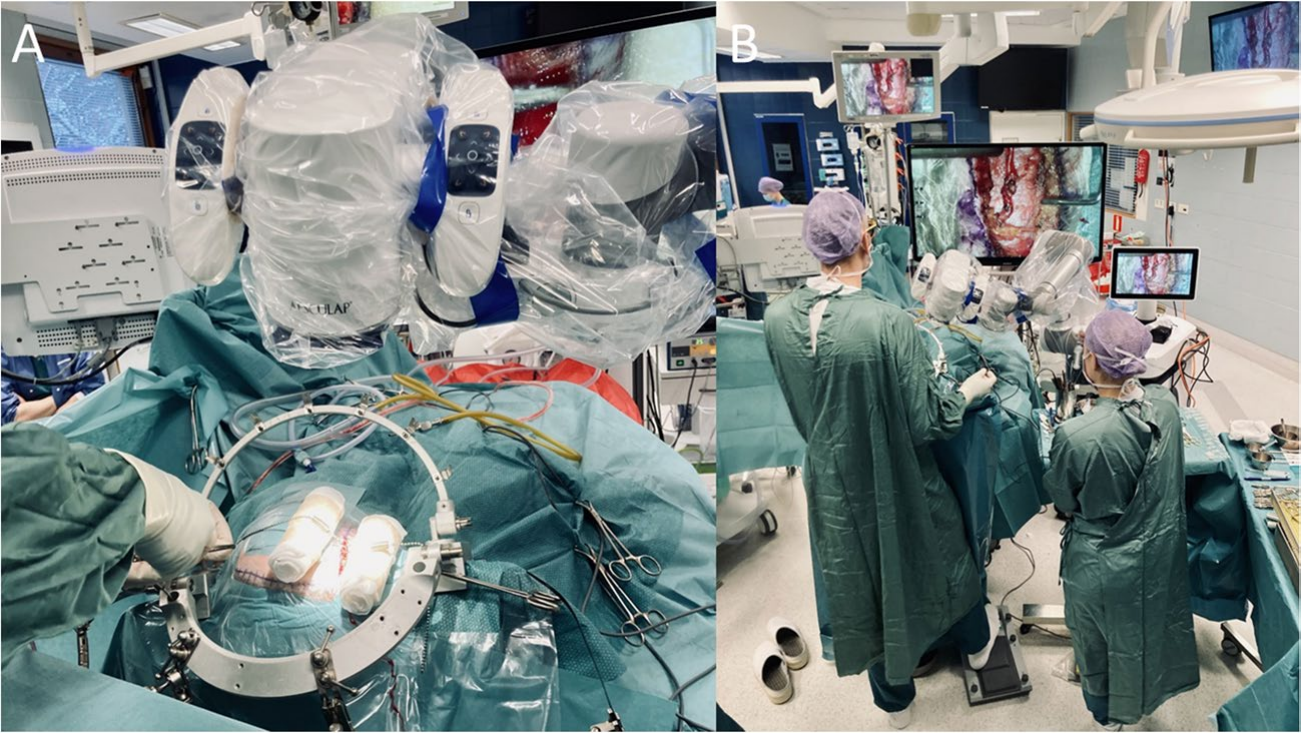
Preparation of the surgical field (**A**) and operative room setup (**B**) for exoscopic STA-MCA surgery. Note the simultaneous foot controlled camera angle adjustment and right hand instrument exchange MCA, middle cerebral artery; STA, superficial temporal artery

The incision is planned during graft (STA) identification by palpating and Doppler sonography. Careful preoperative study of external carotid artery angiograms helps in assessing the location of the STA bifurcation and the size and usefulness of its branches. The incision is planned on top of the main STA trunk (usually the parietal branch) and it’s terminal branches, but may vary depending on anatomy and what is needed. The entire procedure from skin incision to closure is performed under either the microscope or exoscope. In exoscopic bypass surgery, the device is positioned next to the patient with the camera head coming between scrub nurse and surgeon, to float above the surgical field. A high-resolution screen is positioned at the foot-end of the operating table in direct line of sight of the surgeon. The operating room setup is demonstrated in Fig. 1.

Dissection of the STA is begun distally so that if the artery is accidentally injured, the procedure can still continue. The skin is retracted upwards using fishhooks over rolled-up swabs functioning as pulleys elevating and everting skin edges (Fig. 1A). The artery is dissected within the loose areolar tissue and small branches are coagulated and interrupted by a plucking motion using short straight bipolar forceps. The dissection continues proximally and the STA bifurcation is identified. Care is taken to preserve all major proximal side branches not used as a main graft, in order not to compromise further spontaneous collateralization. The main trunk is dissected free to allow mobilization of the vessel. A second dissection run is performed along the vessel, this time from proximal to distal, disconnecting the deep surface of the donor vessel from its bed. The donor vessel is than wrapped in surgical patties soaked with papaverine and pulled gently away with a large piece of latex free surgical glove.

The incision of the temporal muscle is tailored to individual anatomy and whether EDMS is planned or not. The muscle is usually split in a craniocaudal fashion, and the edges are reflected laterally with fishhooks. A single burr hole is made at the cranial border of the planned craniotomy after which a circular craniotomy is completed. Care is taken to protect the graft at all times. The durotomy is placed in such a fashion, that as few dural vessels are transected or coagulated. All potential candidate recipients within the exposed portion of the sylvian fissure are carefully inspected. The recipient vessel is chosen based on size, location and orientation. 

After opening the arachnoid and dissection of the recipient, the donor is clamped and distally disconnected. The STA is cut to length allowing it to be sutured into the recipient’s wall without traction, twisting or looping. The artery is rinsed with heparinized saline and the tip is colored with a water-based surgical marker. The vessel’s end is brought to the anastomosis site and a fish mouth cut is made at the appropriate position. The length of the planned arteriotomy is marked on the recipient vessel wall. The heel- and toe-stiches are preloaded into the donor to shorten ischemia time. Two mini temporary clips are placed at both ends of the recipient starting ischemia time. A straight arteriotomy is made and the donor is firstly fixated with heel- and toe-stiches. Interrupted 10 − 0 nylon stiches are placed to fixate the wings of the fish mouthed donor’s end, pointing away from the surgeon. The wall facing away is sutured first to allow inspection into the anastomosis. The anastomosis is completed by closing the section closest to the surgeon beginning with the wing of the fish mouth. Temporary clips are opened and the anastomosis is inspected for leakage. Some oozing is tolerated and observed under continuous irrigation and augmentation with Surgicel™. In case of relevant leakage, additional stiches are added.

During closure, attention is paid not to kink or twist the donor vessel. The bone flap is trimmed down in size, allowing basal intracranial entry of the STA. Meticulous subcuticular closure aims not to stretch or injure the underlying donor vessel.

### Radiological and clinical evaluation

**Fig. 2:**
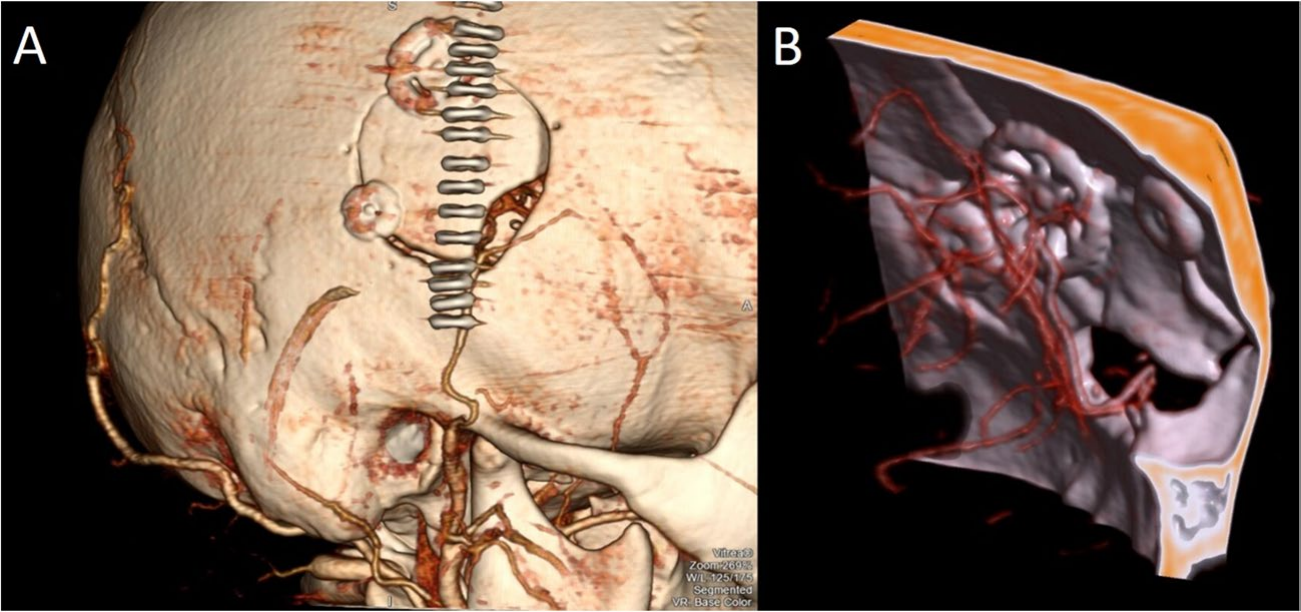
Bypass patency is evaluated in thin slice CT angiography with the aid of 3D rendering techniques (**A** & **B**) 3D, three dimensional; CT, computed tomography

All data were collected retrospectively by screening of electronic hospital records extracting demographic data, symptoms leading to initial MMD diagnosis, comorbidities on admission and occurrence of perioperative complications. Pre- and postoperative imaging was assessed by two independent assessors (MV, VN) including donor diameter (mm.), Suzuki staging [20], direct post-operative bypass patency in CTA imaging and bypass patency at last available follow-up imaging (either CTA or conventional cerebral angiography) (Fig. 2).

### Analysis of surgical procedures and outcome assessment

All surgical procedures were digitally recorded and video material was independently analyzed by two neurosurgeons (MV and RH). Total length of surgery (min.) was chosen as the primary outcome parameter, starting from skin incision until dural closure. Skin closure was not included in total duration of surgery because this was not recorded for all case. Additionally, the total duration (min.) of end-to-side anastomosis was measured (ischemia time) between placing and removing temporary clips on the donor vessel. In one microscopic case, a double-barrel bypass using both main STA branches was performed. In order to include this case, the duration of the first bypass anastomosis was used for analysis of ischemia time and the duration of the second anastomosis was subtracted from the total duration of surgery. Secondary endpoints were defined as: functional outcome measured by the modified Rankin scale [22] (mRS) at discharge and last follow-up, number of stiches placed per anastomosis, number of added stiches after leakage test (as an indirect early measure of bypass quality), bypass patency on direct post-operative imaging and bypass patency on last available imaging.

### Statistical analysis

All descriptive data are presented as mean and (±) standard deviation for normally and as median and interquartile range (Q_1_ to Q_3_) for non-normally distributed continuous variables. Categorical data are presented as proportions. Data was tested for normality via the Shapiro-Wilk test after which the appropriate statistical test was selected. Categorical data were tested by means of the χ^2^ test and continuous data via the unpaired t-test or Mann-Whitney U-test. All statistical analyses were performed using IBM SPSS Statistics 29 (SPSS Inc., Chicago, IL, USA). Statistical significance was defined as a two-sided *p* < 0.05.

## Results

### Patient population and presenting symptoms

In total, 16 consecutive moyamoya patients were included who underwent 21 STA-MCA bypass procedures. The average age of treated patients was 34 ± 11 years (range: 14–55) and proved comparable between microscopic and exoscopic patients. Five patients were treated with staged bilateral bypass surgery. One pediatric patient was included (14 years old) who underwent a combined left sided direct bypass and EDMS, and right direct bypass surgery during a second surgery. Of all included 16 patients, 6 (37.5%) were operated under the operating microscope and 10 (62.5%) using an exoscopic system (ORBEYE® *n* = 1; AEOS® *n* = 9).

The left hemisphere was more commonly and more severely affected in all but two included patients showing left MCA stenosis to some extent. Baseline characteristics and angiographic MMD severity (affected vessels and Suzuki stage) were comparable between microscopic and exoscopic treated patients (see Table 1).

**Table 1:** Baseline- and moyamoya-specific patient characteristics of all included patients (*n* = 16)

	All (*n* = 16)	Microscope (*n* = 6)	Exoscope (*n* = 10)	*p*-value
**Demographics**				
Age - yrs. - mean ± SD	34.2 ± 11.1	34.8 ± 12.3	33.8 ± 11.1	0.865
Sex - Female / Male - no. (%)	11 (68.8 ) / 5 (31.3 )	4 (66.7 ) / 2 (33.3)	7 (70.0) / 3 (30.0)	0.889
**Comorbidities - no. (%)**				
Hypertension	4 (25.0)	0 (0)	4 (40.0)	0.074
Hyperlipidemia	5 (31.3)	1 (16.7)	4 (40.0)	0.330
Diabetes	5 (31.3)	1 (16.7)	4 (40.0)	0.330
Smoking	5 (31.3)	3 (50.0)	2 (20.0)	0.210
**Presenting symptoms - no. (%)**				
TIA	10 (62.5)	4 (66.7)	6 (60.0)	0.790
Stroke	6 (35.7)	2 (33.3)	4 (40.0)	0.790
Hemorrhage	0	0	0	n/a
**MMA Characteristics - no. (%)**				
Left ICA patent	5 (31.3)	2 (33.3)	3 (30.0)	0.889
Right ICA patent	9 (56.3)	4 (66.7)	5 (50.0)	0.515
Left MCA stenotic	14 87.5)	6 (100)	8 (80.0)	0.242
Right MCA stenotic	12 (75.0)	4 (66.7)	8 (80.0)	0.551
Bilat. involvement	13 (81.3)	4 (66.7)	9 (90.0)	0.247
**Suzuki stage**				0.587
Stage 2	6 (37.5)	3 (50.0)	3 (30.0)	
Stage 3	8 (50.0)	2 (33.3)	6 (60.0)	
Stage 4	2 (12.5)	1 (16.7)	1 (10.0)	
Median - (Q1 - Q3)	3.0 (2.0–3.0)	2.5 (2.0–3.25)	3 (2.25–3.00)	0.633

ICA internal carotid artery, MCA middle cerebral artery, Q_1_ first quartile, Q_3_ third Quartile, SD standard deviation, yrs. years

Significant results (*p* < 0.05) are marked in bold

### Duration of surgery and STA-MCA anastomosis

**Fig. 3:**
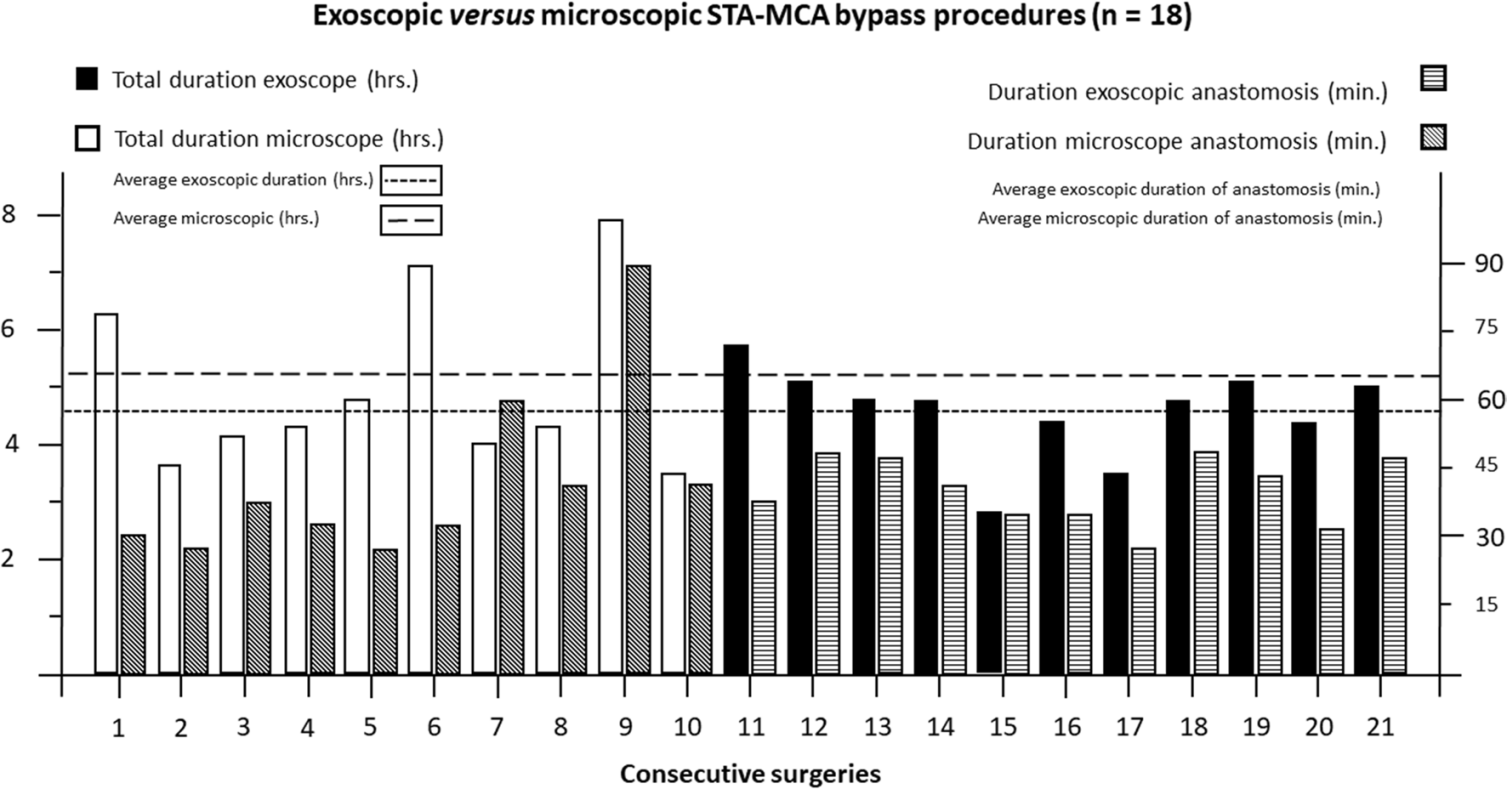
Histogram plotting the duration of surgery and anastomosis (ischemia time) for individual procedures. All first 10 bypass procedures were performed under the microscope, subsequent eleven bypasses were done using the exoscope. Please note that the left y-axis (and duration of surgery) uses hours and the right axis (duration of anastomosis) uses minutes as measure of time hrs., hours; MCA, middle cerebral artery; min., minutes; STA, superficial temporal artery

Total duration of surgery was comparable between microscopic and exoscopic bypass procedures (313 min. ± 116 vs. 279 min. ± 42; *p* = 0.647), ischemia time also proved similar (microscope: 43 min. ± 19 vs. exoscope 41 min. ± 7; *p* = 0.701). The number of individual stiches per anastomosis did not differ between visualization devices (microscope: 17 ± 4 vs. exoscope: 17 ± 2; 0.887). In contrast, more additional stiches were needed in microscopic anastomoses after leakage testing of the bypass (*p* = 0.035). An overview of procedure specific characteristics is presented in Table 2. The duration of surgery and anastomosis is graphically plotted for individual procedures in Fig. 3.

**Table 2:** Overview of procedure-specific parameters and radiological patency of individual bypass surgeries (*n* = 21)

	All (*n* = 21)	Microscope (*n* = 10)	Exoscope (*n* = 11)	*p*-value
Surgical parameters				
Angiographic STA diameter (mm)	1.05 ± 0.25	0.98 ± 0.24	1.12 ± 0.25	0.251
Duration anastomosis (min) - mean ± SD	42.1 ± 13.7	43.3 ± 19.1	40.9 ± 6.7	0.468
Number of stiches	17.2 ± 2.8	17.2 ± 3.6	17.2 ± 1.9	0.918
Number of additional stiches	0.81 ± 1.2	1.4 ± 1.4	0.27 ± 0.6	**0.035**
Total duration (min) - mean ± SD	294.9 ± 84.9	312.8 ± 115.7	278.6 ± 41.8	0.654
**Radiological outcome - no. (%)**				
Bypass patent – early post op	19 (90.5)	9 (90.0)	10 (90.9)	0.943
Bypass patent - last available imaging	19 (90.5)	9 (90.0)	10 (90.9)	0.943

mm, millimeters; min, minutes; SD, standard deviation; STA, superficial temporal artery

Significant results (*p* < 0.05) are marked in bold

### Clinical and radiological outcome

Nineteen out of 21 bypasses were patent on early post-operative imaging with one occlusion in both the microscopic and exoscopic group (*p* = 0.943) (see Table 2). No post-operative ischemia was observed after any of the 21 bypass procedures. No patients experienced a drop in mRS after surgery. During follow-up of a median six months (2.0 to 27.8), no additional bypasses occluded. Caused by the natural progression of the disease, five patients suffered new TIAs during follow-up, of which three in the microscopic and two in the exoscopic group (*p* = 0.210). One cerebral infarction occurred in an patient in the exoscope group (*p* = 0.424). Functional outcome, neither at discharge nor at the latest time point of follow-up, differed between both groups (see Table 3).

**Table 3:** Overview of clinical outcome of included patients (*n* = 16)

	All (*n* = 16)	Microscope (*n* = 6)	Exoscope (*n* = 10)	*p*-value
**Outcome - no. (%)**				
Post-op ischemia	0	0	0	n/a
New TIA during later follow-up	5 (28.6)	3 (50.0)	2 (20.0)	0.210
New stroke during later follow-up	1 (6.3)	0 (0)	1 (10.0)	0.424
mRS - discharge - no. (%)				0.392
no symptoms	7 (43.8)	3 (50.0)	4 (40.0)	
no significant disability	6 (37.5)	2 (33.3)	4 (40.0)	
slight disability	1 (6.3)	1 (16.7)	0	
moderate disability	2 (12.5)	0	2 (20.0)	
mRS - drop pre- vs. post-surgery	0	0	0	n/a
mRS - last follow-up - no. (%)				0.392
no symptoms	7 (43.8)	3 (50.0)	4 (40.0)	
no significant disability	6 (37.5)	2 (33.3)	4 (40.0)	
slight disability	1 (6.3)	1 (16.7)	0	
moderate disability	2 (12.5)	0	2 (20.0)	
mRS - drop since discharge	0	0	0	n/a
Duration follow-up (months)	6 (2.0–27.8)	38.0 (14.0–55.3)	3.0 (1.0–8.5)	**0.007**

mRS, modified Rankin scale; TIA, transient ischemic attack

Significant results (*p* < 0.05) are marked in bold

## Discussion

With this study we aimed to document the performances of a foot switch-operated robotic 3D exoscope system, compared with the conventional operative microscope, for STA-MCA bypass surgery. In this single-surgeon series neither overall duration of surgery nor ischemia time were affected by the visualization device used. A significant lower number of added stiches after leakage testing after exoscopic anastomosis, might suggest the exoscope allows for a better 360 degree assessment during suture placement and better final quality of anastomosis. Although the senior author had bypass experience prior to this series, a learning curve effect herein cannot be excluded.

To the best of our knowledge, this is the third report of exoscopic EC-IC bypass surgery and the first offering a control group. Nossek et al. describe their experience using the ORBEYE Exoscope (Sony Olympus Medical Solutions Inc, Tokyo, Japan) for STA-MCA bypass in five patients, four of which suffered MMD and one patient with a dissecting MCA aneurysm [16]. No exoscope related problems or complications were reported. In addition, Patel et al. reported the use of a 3-dimensional exoscope to perform an internal maxillary to middle cerebral artery high-flow bypass [17].

All exoscope systems on the market today have their advantages and disadvantages. Although the ORBEYE system allows foot switch control of focus, zoom and translational movements, rotational viewing angle adjustments need to be done manually, which can be challenging under high magnification [10]. In an experimental bypass setting, we have previously documented similar duration and quality of anastomosis between microscopic and exoscopic visualization devices [9]. However, the exoscope offers a better 3-dimensional view with increased focal depth alongside the ability to share the surgeons view with others for teaching purposes.

Microvascular intracranial anastomosis in the context of MMD is microneurosurgery *par excellence* and requires optimal magnification and illumination. Unconvincing results of randomized EC-IC bypass trials for atherosclerotic disease have reduced the bypass case load and exposure of neurovascular surgeons to the procedure [7, 18]. In addition, endovascular techniques have reduced the need for revascularization procedures for complex aneurysms [2]. Direct bypass surgery for those remaining MMD cases are oftentimes challenging due to brittle vessels and small pediatric anatomy [5].

We believe surgical exoscopes as microsurgical magnification and illumination devices, might offer better teaching platforms for aspiring bypass surgeons because assistants / residents and OR observers all share the same image as the surgeon. Exoscopes could contribute in tackling the educational paradox of decreasing case load and increasing case complexity [16].

Microvascular bypass surgery requires high magnification and different viewing angles, especially during the actual anastomosing of vessels. The exoscope offers the benefit of additional digital zoom and precise robotic changes of viewing angles under high magnification around a central focus point. Camera adjustments can be performed using a foot-switch, eliminating the need of the surgeon to remove a hand out of the surgical field. Although, foot-switch controlled motorized translational movements along the XY-axes are possible in operating microscopes, hands free rotational movements are not. In combination with increased focal depth, these advantages of exoscope surgery have the potential to shorten the duration of longer procedures that require multiple viewing angle adjustments [21].

Many reports of exoscope use in neurosurgery have remained descriptive and focus on the perception of image quality and improved ergonomics [6, 15]. Here, we aimed at quantifying the transition process from STA-MCA bypass surgery using the binocular operative microscope to the use of a 3D exoscope system. Although there is still much to improve upon, we believe the first generation of exoscopes is capable of allowing complex neurosurgical producers such as neurovascular bypass surgery. These results our congruous to previous reports of exoscope cerebrovascular bypass surgery [16, 17].

### Limitations

As this is a single surgeon consecutive series, the external validity of our results might be limited. On the other hand by comparing results of a single neurosurgeon, smaller incremental changes and improvements in surgical technique as part of a lifelong continuous learning curve aside, other potential sources of biases related to indication, and patient selection, have stayed fairly constant throughout this series. From this point of view, the validity of our results can been graded as good for the purpose of comparing both visualization devices.

Although numbers of included patients are low, to the best of our knowledge, this constitutes the largest series of exoscopic bypass procedures published to date. Bypass patency was, however, not routinely assessed by conventional cerebral angiography (but with CTA) as is common in some institutions. Duration of follow-up varies widely and is relatively short. Also, because exoscopic patients were the last to be operated on, the exoscopic group has a shorter duration of follow-up. This series documents the transition from microscopic to exclusively exoscopic bypass surgery. Although the senior author had extensive prior experience with bypass procedures, a potential learning curve effect cannot be excluded. Other critical microneurosurgical steps such as graft preparation, could have been compared in time and quality, between series. We, however, focused on duration of the anastomosis as this is a fairly standardized part of the surgery and the duration of this step (ischemia time) has relevant clinical implications.

## Conclusion

End-to-side bypass procedures for moyamoya angiopathy using a foot switch-operated 3D exoscope proved safe and leads to comparable clinical and radiological results as classical microscopic bypass surgery. Hands free camera mobility of the exoscope allows the surgeon to keeps both hands in the surgical field during the anastomosis whilst adjusting viewing angles, which might offer the potential to reduce ischemia time.

## Supplementary Information

Below is the link to the electronic supplementary material.

Supplementary file1 (MP4 390 MB)

## Data Availability

The raw data of this analysis can be made available by the authors to any qualified researcher.

## Abbreviations

3D: three dimensional
CT: computed tomography
CTA: computed tomography angiography
EDMS: encoephao-duro-arterio-myo-synangiosis
MCA: middle cerebral artery
min.: minutes
mm: millimeter
MMD: moyamoya disease
SD: standard deviation
STA: superficial temporal artery
TIA: transient ischemic attack
*vs.*: versus
